# Clinical Presentation and Serologic Response during a Rabies Epizootic in Captive Common Vampire Bats *(Desmodus rotundus)*

**DOI:** 10.3390/tropicalmed5010034

**Published:** 2020-03-01

**Authors:** Elsa M. Cárdenas-Canales, Crystal M. Gigante, Lauren Greenberg, Andres Velasco-Villa, James A. Ellison, Panayampalli S. Satheshkumar, Lex G. Medina-Magües, Richard Griesser, Elizabeth Falendysz, Ignacio Amezcua, Jorge E. Osorio, Tonie E. Rocke

**Affiliations:** 1 Department of Pathobiological Sciences, School of Veterinary Medicine, University of Wisconsin-Madison, Madison, WI 53706, USA; crdenascanal@wisc.edu (E.M.C.-C.); medinamagues@wisc.edu (L.G.M.-M.); jorge.osorio@wisc.edu (J.E.O.); 2 Poxvirus and Rabies Branch, Division of High-Consequence Pathogens and Pathology, National Center for Emerging and Zoonotic Infectious Diseases, Centers for Disease Control and Prevention, Atlanta, GA 30333, USA; lzu1@cdc.gov (C.M.G.); foe2@cdc.gov (L.G.); dly3@cdc.gov (A.V.-V.); hio6@cdc.gov (J.A.E.); xdv3@cdc.gov (P.S.S.); 3 Wisconsin State Laboratory of Hygiene, Madison, WI 53718, USA; richard.griesser@slh.wisc.edu; 4 US Geological Survey, National Wildlife Health Center, Madison, Wisconsin, WI 53711, USA; efalendysz@usgs.gov; 5 Comité Estatal para el Fomento y Protección Pecuaria de San Luis Potosí, San Luis Potosí 78310, Mexico; ignacio_murcio@hotmail.com

**Keywords:** rabies virus, outbreak, vampire bat, clinical signs, neutralizing antibody

## Abstract

We report mortality events in a group of 123 common vampire bats (*Desmodus rotundus*) captured in México and housed for a rabies vaccine efficacy study in Madison, Wisconsin. Bat mortalities occurred in México and Wisconsin, but rabies cases reported herein are only those that occurred after arrival in Madison (n = 15). Bats were confirmed positive for rabies virus (RABV) by the direct fluorescent antibody test. In accordance with previous reports, we observed long incubation periods (more than 100 days), variability in clinical signs prior to death, excretion of virus in saliva, and changes in rabies neutralizing antibody (rVNA) titers post-infection. We observed that the furious form of rabies (aggression, hyper-salivation, and hyper-excitability) manifested in three bats, which has not been reported in vampire bat studies since 1936. RABV was detected in saliva of 5/9 bats, 2–5 days prior to death, but was not detected in four of those bats that had been vaccinated shortly after exposure. Bats from different capture sites were involved in two separate outbreaks, and phylogenetic analysis revealed differences in the glycoprotein gene sequences of RABV isolated from each event, indicating that two different lineages were circulating separately during capture at each site.

## 1. Introduction

Rabies is an ancient infectious disease with one of the highest case fatality rates, causing tens of thousands of human fatalities and millions of cattle deaths annually, worldwide [1]. Since the elimination of canine rabies from most of Latin America, the common vampire bat (*Desmodus rotundus*) has become the primary rabies virus (RABV) reservoir. Vampire bat-associated rabies is a major burden to the livestock industry and a significant public health concern across the tropical and subtropical Western Hemisphere [1,2,3,4]. Our research groups at University of Wisconsin—Madison (UW) and U.S. Geological Survey (USGS) National Wildlife Health Center (NWHC) have been developing a recombinant rabies vaccine for use in bats [5,6]. The vaccine candidate is composed of raccoon pox (RCN) as a viral vector for a mosaic gene expressing rabies glycoprotein (MoG) and has conferred protection against rabies challenge in laboratory studies in the big brown bat (*Eptesicus fuscus*) when delivered orally and topically [6]. Given the importance of vampire bats in transmitting rabies in Latin America, the next step in our research was to test the efficacy of the RCN–MoG vaccine candidate in *D. rotundus*. We captured wild vampire bats in the state of San Luis Potosí, located in central México, where rabies is endemic in the southern part of the state [7], and transported them to NWHC in Madison, WI, USA.

The main route of RABV transmission to naive animals is by direct inoculation (e.g., bites) inflicted by a rabid host [1,2]. Vampire bats aggregate in large colonies and are highly social, facilitating RABV transmission to conspecifics [8]. Additionally, the virus itself can drive changes in the behavior of infected animals, allowing for an increased probability of transmission to others. It has been demonstrated that vampire bats excrete RABV in their saliva during late stages of infection and prior to death [9,10]. Previous research has also suggested that infected bats can shed the virus and later recover from the disease, thus suggesting a carrier state is possible [11]. However, this hypothesis was not supported by later experiments [9]. The natural progression of rabies in the vampire bat and its immune response to RABV has not been described in sufficient detail. During the time of capture and temporary housing in México, and also during transport to and final housing at NWHC, numerous vampire bats died, and RABV was detected in their brains. Here, we describe the course of the infection, presentation of clinical signs, and changes in serologic status of captive vampire bats that succumbed to natural RABV infection during captivity prior to experimental activities.

## 2. Case Report

### 2.1. Vampire Bats and Capture Sites

**Figure 1:**
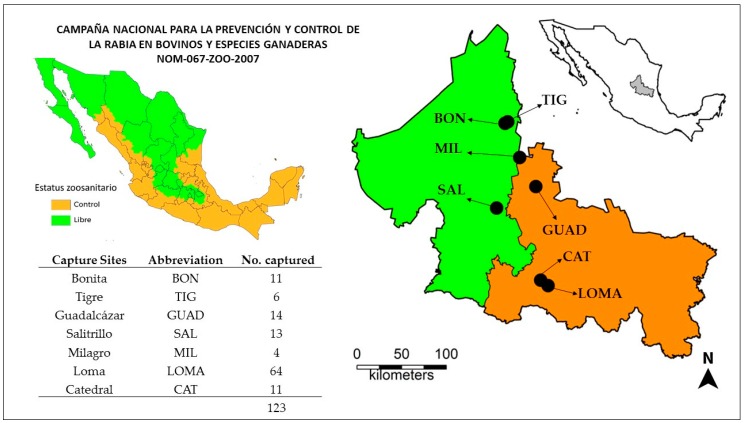
Location of seven capture sites in the State of San Luis Potosí in relation to areas recognized as free (green) or under control (orange) for rabies in livestock species according to the Mexican National Service for Agrifood Health, Safety and Quality (SENASICA) in 2018.

A total of 123 common vampire bats were captured from seven sites throughout San Luis Potosí (Figure 1) during a 6-week period in the summer of 2018 (Jul–Aug). The majority of the bats (n = 75) were captured in the two southern capture locations (Loma and Catedral), where rabies had been reported during activities conducted by the Mexican national campaign for prevention and control of rabies in livestock [7,12]. The remaining bats came from the other 5 locations throughout the state.

We captured bats during the night using mist nets placed at cave entrances, natural or man-made roosting areas (e.g., abandoned water well), around livestock corrals, or known flight pathways. We focused on capturing males and did not collect visibly pregnant or lactating females. Sex and age category (juvenile, adult) were judged by development of external reproductive organs and body size. Following field health inspection, bats were housed temporarily in a room within an animal facility of the Universidad de Matehuala, School of Veterinary Medicine, in Matehuala, San Luis Potosí, until transport to Madison, Wisconsin. The animal room had natural light and ventilation, and humidity was manipulated daily with humidifiers. Bats were grouped by sex and location of origin in mesh cages and were kept on a diet of citrated beef blood and water *ad lib*.

Most bats were held in captivity in México for 4–6 weeks, but the last group captured was held for only 1-week prior to departure. On September 4, 2018, a total of 93 bats (23 females and 70 males) were transported to Madison, Wisconsin, in custom-made cages built in compliance with biosafety and transport guidelines. During the 2-day travel time, bats were fed once with citrated bovine blood at night.

Upon arrival at the NWHC, bats were placed in a biosafety level 3 animal room with controlled temperature (28–34 °C) and humidity (>40%). The bats were combined into new cages of up to 20 animals per cage, but separated by sex and by site of capture, as availability of cages permitted. Husbandry practices remained similar to those in México, and all animal procedures for transport and captivity were approved by the NWHC animal care and use committee under appropriate permits as listed below. After arriving at the NWHC, bats were given an acclimation/quarantine period time of 104 days prior to the experimental vaccination.

### 2.2. Rabies Serology

We obtained an initial (baseline) plasma sample from 88 available bats within days 34–57 after arrival at the NWHC, and periodically thereafter, to determine rabies virus neutralizing antibodies (rVNA). Baseline samples were sent to the Centers for Disease Control and Prevention (CDC, Atlanta, Georgia) and were tested using the micro-neutralization test, a modified version of the Rapid Fluorescent Focus Inhibition Test (RFFIT). Subsequent samples were tested at the NWHC following the same micro-neutralization protocol [13]. When possible, at death, serum was collected as a terminal sample for serology. Evidence of neutralization in >50% of the fields at a 1:10 dilution of the test serum was considered positive for rVNA (or equivalent to reciprocal titers ≥ 1:13). Intermediate samples were those at the cut off for 50% neutralization (i.e., reciprocal titers of 1:10). International units (IU/mL) were calculated as per the protocol; but, given the inherent variability of the test and the lack of a cut off value for wildlife species, we opted to assess serological response based on reciprocal titers and evidence of neutralization [13,14].

### 2.3. Rabies Testing, Typing and Phylogenetic Analysis

In México, a subset of dead bats was sent to the rabies reference laboratory in the city of San Luis Potosí (SPHL) for rabies diagnosis by direct antigen detection (DFA) following the official Mexican standard for rabies diagnosis [12]. In Wisconsin, all dead bats were sent to the Wisconsin State Laboratory of Hygiene (WSLH, Madison, Wisconsin) where brain impressions were tested using the DFA for detection of nucleoprotein antigen following the national standard protocol [15]. Brain samples tested at the WSHL were later submitted to CDC for molecular diagnostic confirmation and sequencing (Appendix A). Sequences were deposited in GenBank under accession numbers MN968374-MN968401. Sequence alignments were generated using MAFFT in Geneious (https://www.geneious.com). Sequence alignments were trimmed to include only the coding region of the nucleoprotein and glycoprotein genes. Maximum likelihood phylogenetic analysis implemented in MEGA software 7.0.26 [16] was conducted by using the GTR+G+I substitution model with 1000 bootstrap replicates to assess the statistical robustness of the tree topology.

We collected oral swabs in RNAlater (Thermo Fisher Scientific, Carlbad, CA, USA) from bats from Loma after the death of 2 bats from that site occurring within a week of arrival at the NWHC. We sent this set of samples to a commercial laboratory for RABV diagnosis using RT-PCR (Zoologix, Inc., Chatsworth, CA, USA). More oral swabs were serially collected from a group of bats involved in a natural outbreak of rabies that peaked in late December. We started swabbing these bats regularly (every 3–5 days) after the outbreak began and for 2 months thereafter. After collection, saliva samples were placed in vials containing 0.5 mL of viral transport media and stored at −80 °C until further testing. These swabs were tested at the NWHC following the LN34 pan-lyssavirus real-time RT-PCR [17,18], with some modifications to the protocol, such as using 8.5 µL of RNA per reaction and using an iCycler instrument (BioRad, Hercules, CA, USA). Cycle threshold (Ct) values ≤ 35 were considered positive (i.e., RABV RNA present), whereas values 35–40 were considered inconclusive and may indicate a low virus load, insufficient sample, or possible cross contamination. RNA extraction was performed using Direct-zol RNA Miniprep kit (Zymo Research, Irvine, CA, USA).

### 2.4. Description of Mortality Events: Suspected and Confirmed for Rabies

In México, bat mortalities were first recorded during capture at the Loma site on Aug 4–5, 2018 (Figure S1 in Supplementary Materials). A total of 64 bats were captured in one night, and 10 bats died before arrival at the animal facility in Matehuala 2 days later. Three of the 10 bats submitted to the SPHL were RABV positive by DFA, and the rest were negative. Trauma or stress during capture and transport were suspected as likely causes of death. During Aug 7–25, 13 bats from Loma, 2 from Guadalcázar, and 1 from Catedral were found dead in their cages. An additional three bats from Loma were euthanized with unhealed wounds that impaired mobility. The last mortality event recorded in San Luis Potosí was on Aug 26, a bat from Catedral that died in good body condition without obvious clinical signs (Figure S1 in Supplementary Materials). Although some bats showed signs (e.g., aggressive behavior and bite wounds) suggestive of rabies infection, testing was not performed, and we cannot assume rabies was the cause of death. A total of 30 vampire bats died while in San Luis Potosí.

Just prior to departure, all surviving animals (n = 93) were given a health check and administered subcutaneous fluids to avoid dehydration while in transit. No signs of disease were observed, and all bats were placed in their correspondent cage for transportation.

**Figure 2:**
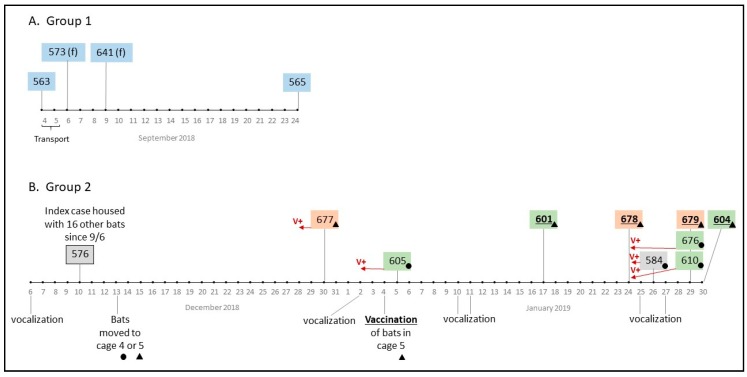
Timeline of vampire bat observations and mortalities recorded in Madison, Wisconsin, from September 2018 to January 2019. All bats were captured in México in August and transported to Madison on September 4–6. Bats from Groups 1 (**A**) and 2 (**B**) were never in contact since capture and presented two distinct RABV lineages based on phylogenetic analysis of N and G gene sequences. Bats in Group 1 with “(f)” are females and were never in contact with males from the same site. Sites of capture are color coded: Loma (blue), Catedral (gray), Guadalcázar (green), and Milagro (orange). Group 2 bats moved to Cage 4 after the first rabies mortality in that group (#576) are indicated with a circle and those moved to Cage 5 with a triangle. Bold/underline lettering indicates bats that were vaccinated. “V+” indicates RABV shedding detected in oral swabs prior to mortality.

During transport, one male bat from Loma (#563, caged with 11 other male bats from the same site) was lethargic and did not survive the 48 h drive to Wisconsin; it tested positive for rabies by DFA at WSLH. On Sept 24, 18 days after arrival at NWHC, one of the 11 bats (#565) died and was also positive for RABV (Figure 2). The other ten bats transported in the same cage showed no signs of rabies. Within a few days of arrival at NWHC on Sept 6, 2018, two of the 10 females (#573 and #641) from Loma died and were found to be RABV positive. One of the females (#641) was euthanized due to an unhealed fracture, and the other bat died suddenly. None of the 3 bats from Loma that died showed signs of rabies prior to death. Bats from Loma were kept separate from all other bats, and no other mortalities occurred in either the males or females from Loma during the observation period presented in this case report.

On Dec 6, 2018, increased vocalization, uneasy behavior, and apparent fighting were recorded in a cage that housed 17 males from Catedral, Milagro, and Guadalcázar. Four days later (day 95 post arrival), Bat #576 (from Catedral) was found dead without prior manifestation of clinical signs and tested positive for RABV (Figure 2). On day 98, bats from this group (n = 16) were split into 2 cages (Cages 4 and 5, Table 1) in preparation for treatment as part of our vaccine study. The next death to occur was Bat #677 from Cage 5 on Dec 30, 2018 (day 115); increased vocalization of the group had been recorded 4 days prior. Bat #677 (from Milagro) showed recent multiple wounds, dehydration, and aggressiveness 2 days before its death. After this death, and because of evidence of aggression within the group, we started collecting oral swabs periodically for RABV testing and bats were examined closely for signs of rabies. One week later (Jan 4, 2019, Day 120) all bats from Cage 5 (n = 10; 5-rVNA negative at baseline, 1-intermediate, 4-positive) were orally vaccinated with RCN-MoG as previously scheduled but also in an attempt to determine if vaccination might stop the outbreak. Bats in Cage 4 (n = 5, all rVNA negative) had received RCN expressing a neutral antigen (luciferase) as a control on Dec 19, 2018.

During Days 121–146, 8 more bats died in Cages 4 and 5 and all tested positive for RABV by DFA. Vocalization was often noticed 3–4 days prior to a new mortality in both México and the United States (Figure 2). Overall, at NWHC 4 bats that succumbed to rabies displayed the paralytic form of the disease: isolation, non-responsiveness, respiratory distress, lethargy, and dull mentation (#584, #676, #601, #679). The furious rabies presentation was observed in three bats only (#677—Milagro, and #605, #610—Guadalcázar), including signs of aggression, hyper-salivation, teeth chattering, irritability to sound and light sources, and excessive vocalization. Eight other bats did not show any clinical signs or evidence of sickness before they were found dead (including #563 that died during transport). In consultation with the NWHC attending veterinarian, bats showing severe distress from either form of RABV infection were euthanized (n = 5).

RABV nucleic acid was detected in oral swabs from 5 (#677, #605, #584, #610, #676) of the 9 rabies-positive bats sampled 2–5 days prior to their death (Table 1). Bat #582 (Cage 4) suffered bite wounds from its cage-mates on Day 115 (one day before RABV was detected in the saliva of Bat #605), yet it did not become ill. An oral swab obtained from Bat #582 on Day 140 was inconclusive for RABV by real-time RT-PCR; all of its remaining samples were negative. The rest of its cage-mates tested positive for RABV in saliva on that same day and eventually succumbed to rabies (Table 1).

All bats that died at NWHC on Days 95–146 (n = 10) were negative for rVNA at baseline; half of these had rVNA at the time of death (Table 2). None of these bats had contact with any of the bats from Loma during capture, transportation, or housing. Surviving bats from Catedral (except #582 and #579) had detectable rVNA at baseline, and titers increased in 5 of the 7 within 25–32 days after the index case died (Table 2). Of the 6 surviving bats that received RCN-MoG, 4 showed an increase in antibody titer 28 days after vaccination.

Vaccination with RCN-MoG had no impact on survival of seronegative bats in Cage 5 as 4/4 vaccinates (#601, #678, #679, and #604) succumbed to rabies, compared to unvaccinated bats in Cage 4 (4/5). However, RABV nucleic acid was not detected in the saliva of 4 vaccinated bats that succumbed to rabies, unlike the saliva samples from unvaccinated individuals (#605, #584, #610, and #676) that were consistently RABV-positive. Seven bats (all from Catedral, one unvaccinated in Cage 4, and six vaccinated in Cage 5) survived up to 231 days, despite being co-housed with bats shedding RABV in saliva, as confirmed by detection of RABV nucleic acid in oral swabs by real-time RT-PCR (Table 1). For the purposes of this case report, the observation or survival period lasted 231 days, when all bats were challenged with a heterologous strain of RABV as part of the vaccine study on Apr 25, 2019.

**Figure 3:**
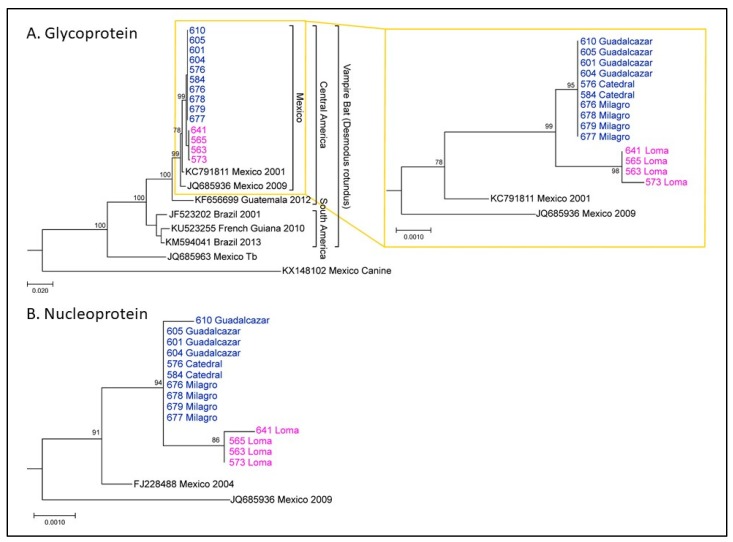
Phylogenetic analysis of glycoprotein (**A**) and nucleoprotein (**B**) nucleotide sequences. Phylogenetic trees were estimated by Maximum Likelihood in Mega7. Newly generated sequences from Mexican *Desmodus rotundus* (magenta, Group 1 and blue, Group 2) clustered with isolates of RABV circulating in *Desmodus rotundus* in Mexico (A, left). The yellow box highlighted on the glycoprotein gene tree is enlarged on the right to show more detail. Numbers at branch points indicate bootstrap value (out of 1000 replicates). Scale bars indicate number of changes per site. Reference sequences from RABV strains circulating in vampire bats in México, Guatemala, Brazil, and French Guiana were included; references from RABV isolated from a Mexican free-tailed bat (*Tadarida brasiliensis*) and a dog (historical) were included as outgroups. Reference sequences are labeled with accession number, country, and year of isolation.

Nucleoprotein (N) and glycoprotein (G) sequences were determined for RABV isolated from brain samples sent to CDC after rabies testing at the WSLH. Phylogenetic analysis revealed that these sequences were consistent with RABV circulating in vampire bats in México. It also revealed two clusters of vampire bat sequences (Figure 3), here called Group 1 and Group 2. Group 1 corresponded to bats from Loma (magenta), which all died shortly after arrival at NWHC. Group 2 corresponded to bats from Catedral, Milagro, and Guadalcázar sites (blue). This lineage showed up at 95 days after arrival at NWHC in a rabid bat (#576) from Catedral. Three Group 1 G sequences were identical, and sample #573 (female) had one change. For Group 1 (n = 4), three sequences had identical N gene sequences and the sample from Bat #641 (female) had one nucleotide change. In Group 2 (n = 10), all G gene sequences were identical, and the N gene sequence was identical for 9/10 samples; one nucleotide change was observed in the sample from Bat #610. Between Groups 1 and 2, there were 4–5 changes throughout the entire coding region of the G gene nucleotide sequence and 2–4 changes in the N gene sequences. These results demonstrate a single lineage introduction likely caused by Bat #576 from Catedral. RABV sequences from subsequent rabid bats co-housed with #576 present the same viral lineage, and all formed a distinctive lineage from those obtained from rabid bats from Loma (Figure 3).

## 3. Discussion

In this report, we describe two separate rabies mortality events that occurred in a group of common vampire bats captured in Central México and transported to NWHC as part of a study to test the efficacy of a recombinant rabies vaccine candidate. We identified mortality events as independent introductions of RABV lineages from bats collected at Loma and Catedral. This was possible by integrating information obtained from a phylogenetic analysis and a detailed mortality timeline, which traced contacts among bats (co-housing history) and potential incubation periods between cases.

Lengthy incubation periods are known to occur after RABV exposure in vampire bats and other species of bats that usually undergo torpor in temperate climates [7,10,19,20]. We observed variable incubation lengths between exposure and rabies confirmation in our captive vampire bats (18 to more than 100 days post primary exposure). Bat #576 (from Catedral), the index case in the rabies mortality event at NWHC, was found to be RABV positive more than three months after bats from Catedral, Milagro, and Guadalcázar were merged into a single cage upon arrival at NWHC. We suspect that the index case or possibly one of the other bats was infected at capture or was exposed to RABV during captivity in México (100–130 days prior to the death of the index case). We also observed shorter incubation periods (18–26 days), similar to those observed in other RABV challenge studies in vampire bats [9,10]. Secondary transmission likely occurred in the event, as we observed bats shedding RABV (e.g., #677 and #605) followed by additional rabies mortalities 20–33 days later (Table 1). We have estimated incubation periods based on the timing between observed events (e.g., mortalities, RABV excretion in saliva samples, and presence of wounds) and exposure to other rabid bats; however, we recognize the lack of sufficient evidence to determine actual incubation periods.

Catedral and Loma sites are located in the southern (rabies endemic) region of San Luis Potosí, whereas Milagro and Guadalcázar are located farther north in areas without or with scarce reports of rabies cases. We observed differences in both survival and clinical presentation after RABV infection between bats from sites considered “rabies-free” and endemic sites. None of the known exposed bats from Guadalcázar and Milagro survived infection, whereas 18/22 Loma bats (males and females combined that arrived at NWHC) and 7/9 from Catedral bats survived co-housing with rabid bats. All bats that died from Guadalcázar and Milagro (n = 8) showed clinical signs (including three furious), whereas bats from endemic sites died without any visible clinical signs. One of the three deaths from Loma (one euthanized female with unhealed wounds) was not suspected of RABV infection and was considered an incidental death. It was somewhat surprising to us that it was positive for RABV by DFA.

The presence of clinical signs in experimentally infected vampire bats is not always observed [21,22,23,24]. In a rabies study with vampire bats, only 47% of the subjects that succumbed to the challenge with different doses of RABV isolated from a cow [21] showed clinical signs, including ataxia, tremors, and paralysis. Mortality and the presence of clinical signs can be dose-dependent [9,19]. The furious presentation of RABV infection in vampire bats has been described in experimental studies in the past [11] but has not been reported in more recent experiments [10,21,22,23,24]. We recorded the aggressive form of the disease (furious) in 3/15 rabies-positive cases in our vampire bats, all from the “rabies-free” sites, but most of the RABV-positive bats had the paralytic form of the disease. Moreover, we detected the presence of RABV nucleic acid in saliva samples of 5/9 bats that succumbed to rabies during the outbreak. Salivary excretion of RABV coincided with the presence of clinical signs. The three bats that presented the furious form of rabies (#677, #605, #610) tested positive for RABV nucleic acid 3–5 days before death in at least one sample tested. We recorded aggressions (i.e., bite wounds) within the groups during days in which salivary excretion of RABV from individuals was confirmed. The presence of vocalization 3–4 days before a death record may indicate a behavioral response from the group (e.g., to avoid or expel a rabid bat from the group). With our limited observations and number of samples, we can only speculate about the rabies transmission history of the bats involved in these mortality events.

Antibody response to RABV and other immune mechanisms (e.g., cell mediated immune response) that drive survival after rabies infection are poorly understood in bats [2,25]. Notably, individuals with pre-existing rVNA did not succumb to rabies infection, although they had known multiple exposures to rabid bats while in captivity. We detected an increased rVNA response in Catedral bats that survived (5/7) approximately 25 days after the start of the second mortality event, which likely represents an anamnestic response [19,21]. Despite being seronegative at baseline, Bat Bat #582 (Catedral) seemed to have an abortive RABV infection that apparently elicited rVNA ~25 days after the first case of rabies occurred that was detected again on day 137 post-outbreak. Conversely, Bat #579 (Cage 5) was negative for rVNA at baseline, and it remained seronegative after vaccination with RCN–MoG and challenge with a heterologous RABV as part of the vaccine study (data not shown). None of the vampire bats that survived showed any indication of disease at any point during the rest of the time in captivity, even those with evident aggression from cage mates during the peak of the outbreak. Previous experiments also report survival of vampire bats after infection with a homologous RABV without the presence of rVNA [22,23], suggesting that some individuals may clear peripheral rabies virus infection by cell-mediated mechanisms [22,26]. Sub-lethal, or abortive, RABV infections have been proposed as a potential explanation for the resistance of bats to rabies, as well as for the sporadic presence of rVNA [19,25,26,27].

As part of our scheduled vaccine study, and in an attempt to halt the ongoing rabies epizootic, we vaccinated bats (n = 10) from Cage 5 on Jan 4, 2019. These bats were collected from the Catedral, Guadalcázar, and Milagro locations, and some had detectable rVNA prior in their initial sample (Table 2). Other investigators have reported that the interaction between a vaccine-induced humoral immune response (neutralizing antibodies) and RABV infection may result in an “early death” phenomenon (i.e., shorter incubation period in vaccinated individuals after infection) [28]. However, this response may also prolong the time to onset of disease [28,29]. A study of foxes vaccinated with a recombinant rabies vaccine after infection with a homologous RABV illustrates these phenomena [29]. Subjects vaccinated early (0 and 3 days post-inoculation, p.i.) had shorter incubation periods than those receiving the vaccine later (14 days p.i.) with all succumbing to the rabies challenge. The range of time to death after vaccination (on Day 120) of our vampire bats was 13–26 days, suggesting an “early death” effect. These bats had been housed with Bat #677 who died on Day 115; RABV was detected in its saliva two days before death. Although our numbers are small for a definitive conclusion, post-exposure vaccination with RCN–MoG did not appear to improve survival. The central nervous system (CNS) of vaccinated animals that died may have already been compromised with RABV infection. Thus, any immune response elicited, regardless of its intensity, would be futile to clear RABV from the CNS, as observed in most rabies cases in mammals [30]. However, vaccination appeared to be associated with suppression of RABV shedding in saliva (Table 1 and Table 2). Future research should focus on corroborating this observation since vaccine-induced suppression of viral shedding in individuals already infected with RABV would help to disrupt the rabies transmission cycle in bat populations.

The genetic differentiation of two RABV lineages circulating within a single State in México was surprising; particularly, considering the close geographic distance between Loma and Catedral (~7 miles). It is likely that the RABV lineage identified in the index case from Catedral (#576) had been recently introduced from a more distant site, since a spillover of such lineage was not observed in bats captured in Loma. Our results indicate a complex dissemination dynamic of RABV associated with vampire bats in San Luis Potosí. More robust sampling is encouraged to further understand these dynamics in wild populations of bats.

The natural transmission dynamics of rabies in vampire bats and their serologic response after exposure to RABV is not entirely understood [2] but certainly has implications for the design and outcome of vaccine efficacy and challenge experiments, in addition to wildlife management strategies. In our captive colony of vampire bats, baseline serology (the absence of rVNA) was a criterion for allocating bats into treatment groups. However, it is possible their rVNA were below detectable limits of the micro-neutralization test, and thus we cannot be certain of their prior exposure history to rabies. The occurrence of a natural rabies epizootic likely caused changes in the immune status of our bats, not to mention the loss of experimental animals. In retrospect, we should have kept bats quarantined for longer periods of time (≥4 months) but given the cost of housing vampire bats in a biosafety level 3 facility as required by 42 CFR § 71.54, that is a difficult proposition. Fortunately, the mortality events at NWHC were confined to bats co-housed in three cages and did not spread to the three other cages in the same room. Our report reveals some of the intricacies of the natural progression of RABV infection in vampire bats and support previous reports on variable incubation periods, presentation of clinical signs, occurrence of abortive infections linked to production of rVNA, and survival after exposure to RABV with no imminent presence of rVNA. In addition, our observations raise questions regarding underlying mechanisms of viral clearance and survivorship of vampire bats to RABV infection.

## Figures and Tables

**Table 1:** Rabies mortality during outbreak in captive vampire bats by capture site in México (Guadalcázar—GUAD; Catedral—CAT; Milagro—MIL). Bats were placed together in a single cage on Day 0 and then split into 2 cages on Day 98 (calendar date indicated above). Oral swabs collected periodically were tested for rabies virus by real-time RT-PCR (Pos = PCR positive, Neg = PCR negative; Inc = inconclusive). Bats that died and were confirmed rabies positive by DFA are indicated (M) on day of death. Clinical signs present at time of death (F = furious, P = paralytic, NS = no clinical signs, - = did not die).

Day after Arrival at National Wildlife Health Center PCR Result on Saliva and Rabies Mortality Confirmed by DFA																
Bat ID	Site of Capture	12/10	12/13	12/28	12/30	1/2	1/4	1/5	1/7	1/9	1/17	1/24	1/26	1/29	1/30	Signs at Death
		95	98	113	115	118	120	121	123	125	133	140	142	145	146	
576	CAT	M	-	-	-	-	-	-	-	-	-	-	-	-	-	NS
605	GUAD	-	To Cage 4	-	-	Pos	-	M, Pos	-	-	-	-	-	-	-	F
584	CAT	-		-	-	Neg	-	-	Neg	-	Neg	Pos	M	-	-	P
610	GUAD	-		-	-	Neg	-	-	Neg	-	Neg	Pos	-	M	-	F
676	MIL	-		-	-	Neg	-	-	Neg	-	Neg	Pos	-	M, Pos	-	P
582	CAT	-		-	-	Neg	-	-	Neg	-	Neg	Inc	-	Neg	-	-
677	MIL	-	To Cage 5 ^‡^	Pos	M	-	-	-	-	-	-	-	-	-	-	F
601 ^†^	GUAD	-		-	-	-	Neg	-	Neg	Neg	M	-	-	-	-	P
678 ^†^	MIL	-		-	-	-	Neg	-	-	Inc	Neg	M	-	-	-	NS
679	MIL	-		-	-	-	Inc	-	-	-	-	-	-	M, Neg	-	P
604	GUAD	-		-	-	-	Neg	-	-	-	-	-	-	Neg	M	NS
579	CAT	-		-	-	-	Neg	-	-	-	-	-	-	Neg	-	-
583	CAT	-		-	-	-	Neg	-	-	-	-	-	-	Neg	-	-
575 ^†^	CAT	-		-	-	-	-	-	Neg	Neg	Neg	Neg	-	Neg	-	-
585 ^†^	CAT	-		-	-	-	Neg	-	Neg	Neg	Neg	Neg	-	Neg	-	-
580	CAT	-		-	-	-	Neg	-	-	-	-	-	-	Neg	-	-
581	CAT	-		-	-	-	Neg	-	-	-	-	-	-	Neg	-	-

^†^ Four bats with signs of bites were moved to a separate cage for closer observation on Day 116. ^‡^ Ten bats in Cage 5 were vaccinated against rabies with a raccoon poxvirus expressing rabies glycoprotein (RCN-MoG) on Day 120.

**Table 2:** Antibody titers of vampire bats collected from sites in México (Guadalcázar—GUAD; Catedral—CAT; Milagro—MIL) involved in natural rabies outbreak at baseline, post-outbreak (pre-vaccination; Days 119–127), post-vaccination (Day 148), and terminal for those bats that died of rabies during the outbreak. Treatments included RCN-expressing mosaic glycoprotein (RCN–MoG), a rabies vaccine candidate, and RCN-luciferase (*luc*), a placebo. Age group is indicated as A = adult, J = juvenile.

Bat ID	Capture Site	Age	Treatment	Baseline	Post-Outbreak	Post-Vaccination	Terminal	Outcome
576	CAT	A	-	<1:10	-	-	<1:10	-
605	GUAD	J	RCN-l*uc*	<1:10	-	-	1:1635	-
584	CAT	A	RCN-*luc*	<1:10	<1:10	-	<1:10	-
610	GUAD	A	RCN-*luc*	<1:10	<1:10	-	<1:10	-
676	MIL	J	RCN-*luc*	<1:10	<1:10	-	1:102	-
582	CAT	A	RCN-*luc*	<1:10	1:158	1:611 ^§^	-	survived
677	MIL	A	-	<1:10	-	-	1:3418	-
601	GUAD	J	RCN-MoG	<1:10	<1:10	-	<1:10	-
678	MIL	J	RCN-MoG	<1:10	<1:10	-	1:38	-
679	MIL	A	RCN-MoG	<1:10	<1:10	-	<1:10	-
604	GUAD	A	RCN-MoG	<1:10	<1:10	-	1:559	-
579	CAT	J	RCN-MoG	<1:10	<1:10	<1:10	-	survived
583	CAT	A	RCN-MoG	1:10	<1:10	1:49494	-	survived
575	CAT	A	RCN-MoG	1:22	1:3418	1:13975	-	survived
585	CAT	A	RCN-MoG	1:22	1:27	1:112	-	survived
580	CAT	A	RCN-MoG	1:18	1:3418	1:2286	-	survived
581	CAT	J	RCN-MoG	1:22	1:559	1:2287	-	survived

^§^ Bat #582 was bled on Day 177.

## Supplementary Materials

The following are available online at https://www.mdpi.com/2414-6366/5/1/34/s1, Figure S1: Timeline of observations and mortalities recorded in San Luis Potosí and Madison, Wisconsin, from August 2018 to January 2019. Sites are indicated CAT = Catedral (gray), LOMA = Loma (blue), GUAD = Guadalcázar (green), MIL = Milagro (red).

## Appendix A

### Sequencing and Analysis

RNA was extracted from rabies-positive brain samples using the Directzol miniprep kit (Zymo). cDNA was generated using random hexamer oligonucleotides and AMV reverse transcriptase (Roche, Sigma-Aldrich, St. Louis, MO, USA). Complete nucleoprotein and glycoprotein coding sequences were amplified using Takara long amplicon taq polymerase (Takara Bio USA, Mountain View, CA, USA) Samples were multiplexed using a 96 barcoding PCR kit (Oxford Nanopore Technologies, Oxford, United Kingdom) and Takara long amplicon TaqLibrary preparation was performed following instructions for Oxford Nanopore Ligation Sequencing Kit 1D (SQK-LSK108). Sequencing was performed overnight on a MinION sequencer using MIN107 R9 SpotON flow cells. Basecalling and demultiplexing was performed using Albacore version 2.3.0 Sequences were aligned to reference rabies virus genomes using bwa mem –x ont2d. Consensus sequences were generated from sam mapping files in CLC genomics workbench version 11 using a minimum threshold of 50× coverage. Consensus sequences were corrected using Nanopolish version 0.10.2 (https://github.com/jts/nanopolish/). The three single nucleotide differences within Group 1 or Group 2 were confirmed by Sanger sequencing.

Single nucleotide insertions and deletions were manually corrected using the following method. Nucleoprotein and glycoprotein coding regions of consensus and polished consensus sequences were aligned to rabies virus reference sequences using mafft v7.308 [31,32] in Geneious 9.1.4 (Biomatters, Inc., Newark, NJ, USA). Alignments were manually inspected for indels relative to the reference sequence. If an indel occurred in only the consensus or the polished corrected sequence, the sequence that had no indel relative to the reference was used. For insertions in a homopolymer, one of the homopolymer bases was removed. For a deletion in a homopolymer, the base to insert was determined by the homopolymer present in the consensus sequence. In cases where indels were detected between two neighboring homopolymers, an ambiguous base was used. When a non-homopolymer nucleotide difference was observed between the consensus and the polished consensus, the nucleotide in the polished consensus was chosen.
