# Supplementary figures and images for: Clinical Presentation and Serologic Response during a Rabies Epizootic in Captive Common Vampire Bats (Desmodus rotundus)
Source: Trop Med Infect Dis. 2020 Mar 1;5(1):34. doi: 10.3390/tropicalmed5010034 (PMC7157733; doi:10.3390/tropicalmed5010034)

### AUGUST 2018

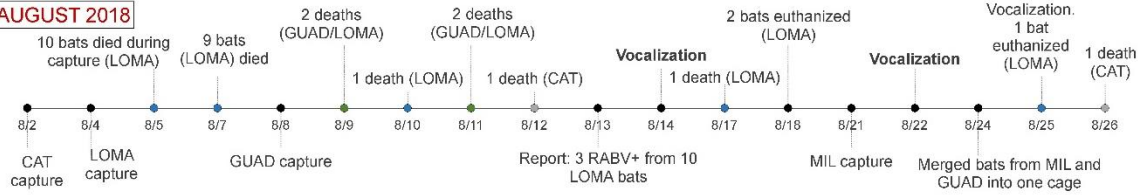

### SEPTEMBER 2018

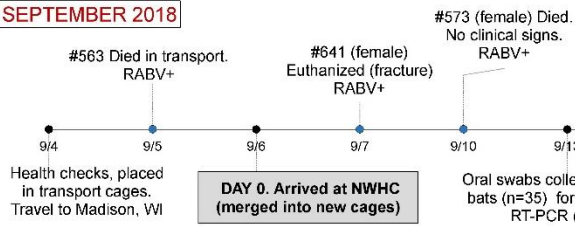

### OCTOBER/NOVEMBER 2018

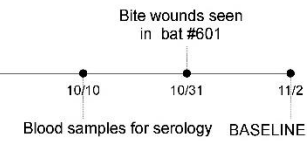

### DECEMBER 2018

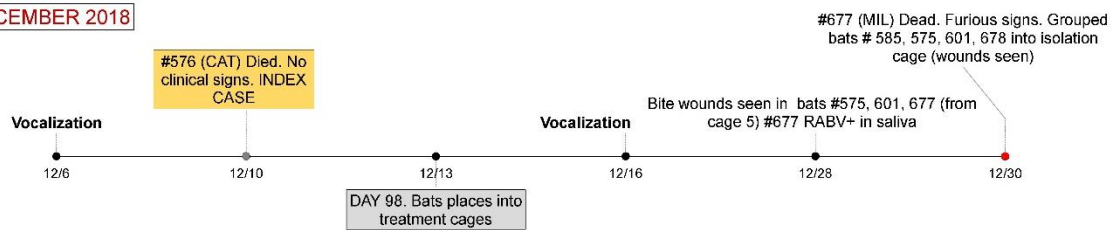

### JANUARY 2019

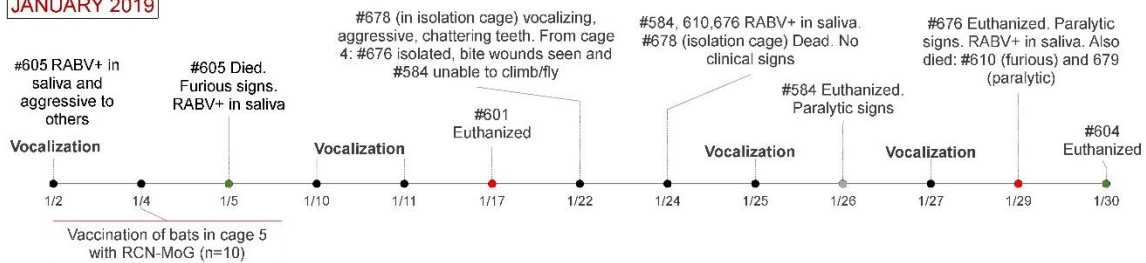

Supplement: Supplementary file 1 [file tropicalmed-05-00034-s001.pdf]
